# Unveiling the Therapeutic Potential of Probiotics in Sepsis: A Review

**DOI:** 10.1002/fsn3.70364

**Published:** 2025-06-08

**Authors:** Zhaopeng Wang, Jiaqi Huang, Peng Zhao

**Affiliations:** ^1^ Medical Department The Second Affiliated Hospital of Harbin Medical University Harbin China; ^2^ Medical Insurance Office The Second Affiliated Hospital of Harbin Medical University Harbin China; ^3^ Department of Ultrasound The Second Affiliated Hospital of Harbin Medical University Harbin China

**Keywords:** diet, gut microbiota, immune response, inflammation, organ failure, probiotics, sepsis

## Abstract

Sepsis continues to be among the most significant causes of mortality and morbidity globally, as defined by an exaggerated host response to infection resulting in a peak of systemic inflammation, organ dysfunction, and unnecessary mortality. The many complexities of sepsis, such as its pathophysiology, current treatments, and its evolving place within nutrition care, are debated. The major etiologies of sepsis are bacterial, viral, and fungal infections leading to an inappropriate immune response with cytokine storm, endothelial damage, and multiorgan failure. Although there has been advancement in medical management, the current treatment approaches, like antibiotic therapy, fluid therapy, and organ support, are insufficient to decrease mortality; therefore, there is a pressing need for effective treatment. Current studies target the central position of nutrition in sepsis treatment, that is, gut microbiota in immune function and systemic inflammation. Nutritional treatment, encompassing enteral and parenteral nutrition, is designed to support metabolic homeostasis, boost immune resistance, and modulate catabolic stress in critically ill patients. The gut‐sepsis axis is also emphasized, since dysbiosis and intestinal barrier dysfunction lead to systemic inflammation and aggravate sepsis outcomes. Recent evidence indicates that probiotics can provide adjunct benefits via gut microbiota manipulation, immune system augmentation, and anti‐inflammatory effects. This review accentuates the requirement for integrative therapeutic strategies in the form of conventional sepsis treatment augmented by specific nutritional interventions to optimize patient survival and recovery. Follow‐up studies must focus on improving individualized nutrition therapy, clarifying the role of gut microbiota in the pathogenesis of sepsis, and finding new therapeutic targets for preventing complications of sepsis.

## Graphical abstract

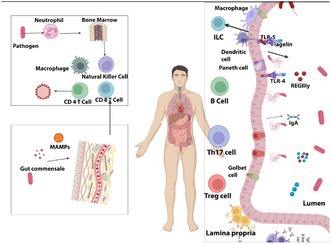

## 1 Introduction

The infiltration of bacteria along with other harmful germs, which may originate from an infection in any area of the body, induces sepsis, a condition known as systemic inflammatory response syndrome (Hotchkiss et al. 2016). One of the most prevalent causes of fatalities in intensive care units is sepsis, which frequently happens to patients with significant illnesses such as severe burns, numerous injuries, postoperative patients, and so forth. It is thought that the pathophysiology of sepsis involves the gut. When it comes to protecting the body from germs and their toxins, the gut barrier is the first line of defense (Adelman et al. 2020). Moreover, patients with sepsis suffer from a systemic inflammatory response syndrome as a result of immunological responses such as oxidative stress and cytokine production that are overstimulated and dysregulated. It is thought that the intestinal microbiome's makeup affects bacterial translocation (BT), gut barrier function, and ultimately systemic inflammation. Sepsis‐related mortality is significant, and the present treatments for the condition are poor. Thus, it is critical to identify novel therapeutic approaches that lower the disease's death rate (Dos Santos et al. 2021). Probiotic use is one of the most promising therapeutic options for sepsis, and numerous studies have documented its positive effects on patients. These are living microorganisms that can benefit their hosts. These beneficial microorganisms primarily consist of Lactobacillus, Bifidobacterium, Streptococcus, Saccharomyces, Bacillus, and Enterococcus (Chen et al. 2019; Guo et al. 2019; Han et al. 2020). Acute pancreatitis, ventilator‐associated pneumonia, sepsis, surgical infections, and diarrhea linked to antibiotics are all treated with these strains. According to an increasing amount of experimental and clinical data, probiotics might enhance intestinal integrity, inhibit BT, minimize the proliferation of pathogenic microbes, conceal the creation of cytokines, and lessen intestinal epithelial cell apoptosis (Angurana et al. 2018; Chen et al. 2020).

Although probiotics can assist with diseases like sepsis, there are potential risks associated with them, particularly for elderly, severely ill, and neonatal patients (Doron and Snydman 2015). Rarely, they have been connected to allergic responses, infections, and even the development of antibiotic resistance. Additionally, vulnerable people may experience problems such as fungemia or bacteremia (Mikucka et al. 2022). Therefore, it is important to consider both positive and negative aspects before usage. To guarantee secure consumption, especially for certain groups of patients, more focused, carefully orchestrated clinical trials are still essential. This review aims to open the door for innovative next‐generation medicines, precision probiotics, and personalized microbiome therapy as treatment alternatives for sepsis patients.

## 2 Sepsis: A Global Health Crisis

Sepsis is one of the leading causes of morbidity and mortality across the world. Sepsis accounted for 48.9 million estimated cases and 11 million deaths in the year 2017, accounting for around 20% of all deaths globally (Rudd et al. 2020). The worldwide prevalence of sepsis is varied and influenced by a number of variables, such as the wellness of the population, changing demographics, and access to medical care. Because of differences in healthcare systems and surveillance, sepsis prevalence varies widely around the globe. Sepsis is hyperendemic among low‐ and middle‐income countries (LMICs) as a result of poor healthcare infrastructure, late diagnoses, and unavailability of antibiotics and intensive care facilities. With an estimated 2.9 million new instances of infection that lead to sepsis‐related fatalities annually, newborns and maternal sepsis are among the primary causes of fatality in the previously mentioned countries (Liu et al. 2014). In conjunction with tuberculosis (TB), which is unfortunately also a pandemic in LMICs, and malaria, sepsis is one of the primary reasons for death for people living with HIV/AIDS (Rudd et al. 2020). The most common causes of sepsis‐related mortality, which usually take place in settings with inadequate facilities, are maternal and neonatal sepsis. South Asia and sub‐Saharan Africa suffer the greatest number of the 2.5 million newborns who die from sepsis each year. Maternal sepsis is a major cause of deaths associated with pregnancy and can be rendered worse by postpartum and delivery infections. A large portion of the world has seen an increase in mortality as a result of advancements in the treatment of severe sepsis, such as early identification, fluid resuscitation, and antibiotics. Furthermore, sepsis bundles from the Surviving Sepsis Campaign have considerably reduced mortality (Rhodes et al. 2017). Inequities in access to care, postponed diagnosis, and resistance to antibiotics continue to be problems in sepsis results globally.

Even in developed nations, it is a huge responsibility, especially in the elderly and chronic patients. High‐income, developed countries have seen an increase in severe sepsis with age. According to a study, 270,000 individuals die and 1.7 million others fall ill in the US every year. Depending on how various individuals record and diagnose the condition, the yearly prevalence of sepsis in Europe might vary between 50 and 300 cases per 100,000 people. Hospital‐acquired infections can contribute to sepsis lethality in a healthcare setting, including pneumonia associated with ventilators and catheter‐induced bloodstream illness. Even after the extensive advancement in medicine, sepsis is a global health issue that takes the lives of millions of people each year. The World Health Organization (WHO) includes sepsis as an international health issue that must be diagnosed earlier, prevented, and controlled (WHO 2020). Sepsis is a worldwide health crisis that affects millions of people every year and profoundly tests worldwide health systems. Early detection in the course, timely treatment, and prevention are necessities that can reduce sepsis morbidity and mortality. In the upcoming years, sepsis biomarkers must be optimized, new therapies must be developed, and sepsis healthcare disparities throughout the world's varied regions must be addressed. There should be more awareness, global health programs, and policy reforms to combat this silent killer.

## 3 Current Therapeutic Limitations (Antibiotics, Fluid Resuscitation, Organ Support)

Pharmaceutical medicines may be used to treat sepsis because bacterial infections account for the majority of sepsis cases. Early and timely therapy with antibiotics has been associated with higher survival rates. Nonetheless, the utilization of medications in sepsis is subject to significant limitations. One of the biggest challenges in treating sepsis is the increasing incidence of antibiotic resistance (AMR). Multidrug‐resistant (MDR) bacteria originate from antibiotic misuse and abuse, making diseases challenging to cure.

Antimicrobial resistance, also known as AMR, is a new global problem that raises mortality and makes treating sepsis more difficult, according to the WHO (2020).

Microorganisms that are more prevalent in sepsis include extensively drug‐resistant *Acinetobacter baumannii* , carbapenem‐resistant Enterobacteriaceae (CRE), and methicillin‐resistant *Staphylococcus aureus* (MRSA). Early intervention with antibiotics is essential for treating sepsis. According to research, the risk of mortality rises with each minute that goes by without initiating the appropriate antibiotic treatment. Initially, empirical broad‐ranging antibiotics are used, but inadequate selection may lead to drug resistance and other unintended consequences, and the time spent on microbiological diagnostics makes it more difficult to identify the causative organism and choose the most effective antibiotic treatment. It is nevertheless difficult to apply antibiotic stewardship techniques in sepsis, even when they are used to maximize antibiotic exposure while lowering resistance. Advanced diagnostic tools and trained staff are needed to maximize a balance between de‐escalation and upfront broad‐spectrum coverage (Rhodes et al. 2017). Fluid resuscitation is crucial in the medical management of sepsis because it improves tissue perfusion and restores intravascular volume. Other areas of disagreement include the type, quantity, and period of the administered fluid. Early and forceful resuscitation with 30 mL/kg of crystalloids has been recommended by the Surviving Sepsis Campaign recommendations, especially during the initial 3 h (Evans et al. 2021).

A recent study found that imbibing too much fluid might result in a surplus of fluid and raise the risk of complications such as acute kidney injury (AKI), pulmonary edema, and a higher need for mechanical ventilation (Marik et al. 2017). There is also disagreement over which fluid is optimal for resuscitation. The best choices are balanced or normal saline solutions because they are affordable and easily accessible. On the other hand, hyperchloremic acidosis and increased AKI have been linked to excessive saline delivery. Although albumin and hydroxyethyl starch (HES) colloids have been investigated, they are related to health and cost issues. Due to the increased risk of renal impairment and mortality, HES should not be considered for use in septic patients (Perner et al. 2012).

Adopting customized fluid resuscitation according to the patient's condition—including the responsiveness of fluids and hemodynamic status—can yield better results, according to an increasing amount of studies. Even while dynamic indications like passive leg raise tests and pulse pressure fluctuation have been studied as tools to direct fluid therapy, they are not yet used in ordinary practice. Severe sepsis requires supportive treatment techniques, such as vasopressor therapy, renal replacement therapy (RRT), and ventilatory support with mechanical ventilation. The best possible organ support optimization is nevertheless hampered by critical care improvements. One serious consequence of sepsis is acute respiratory distress syndrome (ARDS), which requires lung ventilation. Evidence suggests that body weight ventilation is successful at a modest tidal volume of 6 mL/kg. Ventilator‐induced lung injury (VILI), on the other hand, is a complication that requires adjunct therapy by prone position and neuromuscular blockade. Sepsis is the leading etiology of AKI and 50% of septic critically ill patients need RRT (Bagshaw et al. 2017). Though continuous RRT (CRRT) is usually the preferred mode in hemodynamically unstable patients, the optimal timing and modality of RRT remain uncertain. Early initiation of RRT has not always been linked with a survival advantage, and delayed initiation can result in augmented renal injury. Hypotension is a characteristic finding of septic shock, which requires vasopressor support to sustain adequate mean arterial pressure (MAP). Norepinephrine is the agent of choice for initial vasopressor, but optimization and timing of augmentation of therapy remain uncertain.

## 4 The Gut‐Sepsis Relation: Pathophysiology and Dysbiosis

### 4.1 Role of Microbiome Alteration in Sepsis Progression

A number of scholars have hypothesized that alterations in the microbiota's composition may put patients in danger for immunosuppression and, ultimately, sepsis, since a healthy gut microbiota has been showed to shield the host and prevent the colonization of bacteria that are tolerant to multiple drugs. Sepsis, a complex medical issue that may outcome in septic shock and even death, is characterized by an uncontrollable inflammatory response to a pathogen (Hotchkiss et al. 2016). The World Health Assembly (WHA), which makes decisions for the WHO, has escalated its quest to avoid, detect, and treat sepsis because it has begun to recognize the condition as an imminent risk to patient health and global wellness (Kim and Park 2018). According to recent studies, sepsis has a direct connection with alterations in the gut microbiota, and the microbiome itself may be the key to comprehending the pathogenesis and prognosis of the condition. The gastrointestinal microbiota, which includes bacteria, viruses, fungi, protozoa, and archaea, is essential to both the growth and function of the intestinal epithelial barrier as well as the immune system that is necessary for the host's long‐term health (Piccioni et al. 2024). The importance of the microbiota in the gastrointestinal tract in both good and bad health has been established. Sepsis‐induced acute dysbiosis of the gut microbiota enables pathobionts to proliferate.

By weakening the intestinal barrier, these alterations may weaken the immune system of the patient and make the disease worse (Hotchkiss et al. 2016). The gut microbiota, a diverse group of microorganisms, is essential for preserving immune and metabolic function.

**FIGURE 1:**
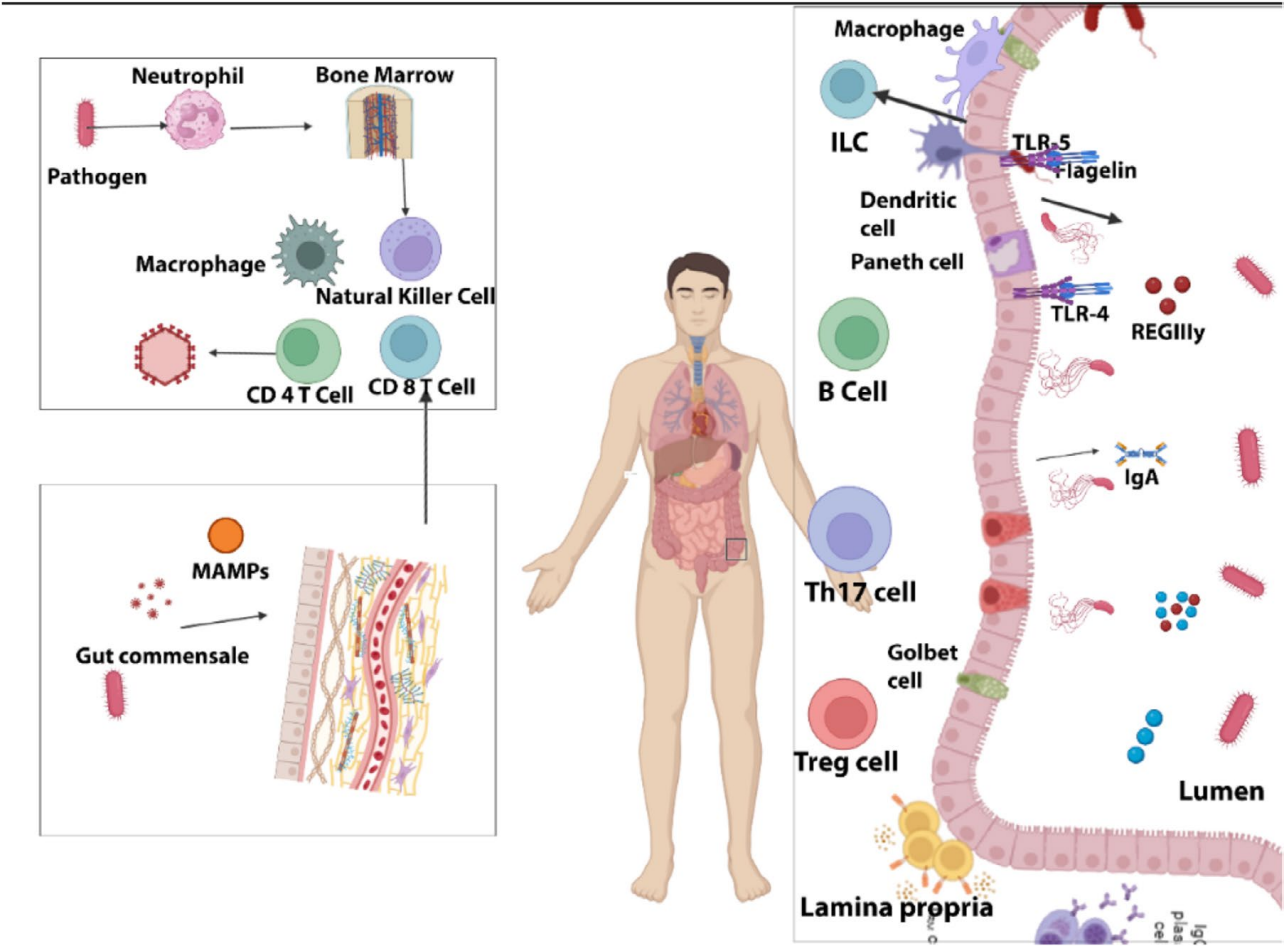
The role of gut microbiota in sepsis management.

Sepsis causes a decrease in the diversity of microbial species and an increase in pathogenic microorganisms, such as proteobacteria, which worsen inflammation (Adelman et al. 2020). According to recent research, an altered gut flora may have an influence on the onset and aggravation of sepsis (Figure 1, Table 1).

**TABLE 1:** Gut microbiota composition in patients with sepsis.

No of participants	Analysis	Microbial composition	Reference
41 sepsis patients	*16S rRNA* gene sequencing	The ratio of Firmicutes to Bacterodites was considerably lower when sepsis was diagnosed	Luan et al. (2024)
Ten sepsis patients and ten non‐sepsis patients and	*16S rRNA* gene sequencing	Both sepsis and non‐sepsis patients had poorer gut flora α‐diversity and structure than the healthy control group. The phylum Firmicutes dominated, with much lower proportions of Bacteroidetes, Prevotella, and Lachnospira, among other taxa. Notably, sepsis patients' digestive tracts had a considerably greater prevalence of Enterococcus	Yang et al. (2021)
20 septic patients and 20 healthy children 155 patients	*16S rRNA* gene sequencing	Microbes closely linked to inflammation, including Parabacteroides, Fusobacterium, and Bilophila species, were more prevalent in the microbiota of sepsis patients During the ICU stay, a notable decline in microbial diversity was also noted. At the same time, sepsis patients who died had a considerably higher quantity of dangerous species, including Enterococcus species, suggesting that these species could be biomarkers	Agudelo‐Ochoa et al. (2020)
25 children with sepsis and 15 age‐ and sex‐matched healthy controls	16S rRNA gene sequencing	Compared to healthy controls, children with sepsis had a substantially less diverse gut microbiome. There were also significant changes in the gut microbiota's overall community structure. On a genus level, opportunistic pathogens like Acinetobacter and Enterococcus were more prevalent in children with sepsis, whereas fewer beneficial bacteria like Roseburia, Bacteroides, Clostridium, Faecalibacterium, and Blautia were found	Du et al. (2021)
14 septic patients	16S rRNA gene sequencing	The prevalent phyla during hospital admissions were Proteobacteria, Firmicutes, and Bacteroidetes. Sepsis patients' gut microbiota diversity drastically declines during intensive care unit stays, and individuals with varying degrees of sepsis have diverse dynamic changes in their gut microbiota	Liu et al. (2023)

### 4.2 Disruption of Intestinal Barrier Integrity

The gut microbiota's capacity to displace infections and generate compounds that control different host processes makes it essential for preserving the integrity of the intestinal barrier. For example, butyrate and propionate bring about the production of proteins such as ZO‐1 and occludin that enhance the cell‐to‐cell junctions. Junctional proteins that preserve barrier integrity encompass claudins, occludins, and cytosolic proteins (Ghosh et al. 2020). A diverse, balanced microbiome minimizes permeability and inhibits harmful substances from entering the bloodstream, boosting the strong linkages between intestinal epithelial cells. Endotoxins (such as lipopolysaccharides [LPS]) and other microbial products are believed to be able to enter the bloodstream through dysbiosis. Translocation risk may rise if the epithelial barrier becomes compromised by an influx of pathogenic bacteria (Puebla et al. 2021).

The composition and activity of the gut microbiota may be influenced by comorbidities, diet, and antibiotic exposure, all of which may have an impact on the possibility of translocation. For instance, antibiotics have the ability to disrupt the normal balance of the microbiota, which can lead to the development of pathogenic bacteria that can penetrate the intestinal barrier (Ghosh et al. 2020). Furthermore, the drugs can significantly alter the microbial ecology, which may end in the formation of highly harmful but often resistant pathogens like Enterococcus fecium and *Klebsiella pneumoniae* .

This is particularly valid for drugs that stop anaerobic responses. The study found that after receiving antibiotic treatment, mice given an obesogenic Western diet (WD) were more susceptible to multiorgan damage and deadly sepsis. A gut microbiota research indicates that WD alone raises Proteobacteria growth, decreases Bacteroidetes, and increases the presence of antibiotic resistance indicators that appear before antibiotics are administered (Hyoju et al. 2019). In another study, the clinical outcomes of 15 high‐risk individuals were associated with their gut bacterial composition. The findings demonstrated that antibiotics substantially altered the general arrangement of the microbiome, primarily increasing the frequency of Enterococcus. The probability of developing antibiotic resistance becomes greater by post‐antibiotic dysbiosis, which is frequently characterized by a loss of taxonomy and functional variation as well as decreased colonization resistance against pathogenic microbes (Lange et al. 2016).

### 4.3 Systemic Immune Dysregulation

BT and the breakdown of the intestinal barrier are important factors in the pathophysiology of sepsis and related organ failure. One way by which bacteria from the intestines might proliferate across the body is by translocation of bacteria. They can also activate the gut immune system or travel through the mesenteric lymph nodes (MLNs). Due to the dissolution of tight connections, bacteria in BT travel by paracellular networks and transcellular passages between enterocytes that are regulated by membrane pumps (Charitos et al. 2024). Physical, physiological, and immunological elements combine to form gut barrier dysfunction, which intensifies these mechanisms. Antigens and endotoxins are moved from the gastrointestinal system into the circulation of the body. Toxic bacterial products, both viable and non‐viable, move around and feed a vicious cycle of systemic inflammation and dysregulated immune activation in sepsis. The pathogen‐induced immune response is convoluted, resulting in a dysregulated innate and adaptive immunological response that can result in immunosuppression, excessive inflammation, and a lack of ability to restore immune balance if it is not swiftly tackled (Piccioni et al. 2024).

**FIGURE 2:**
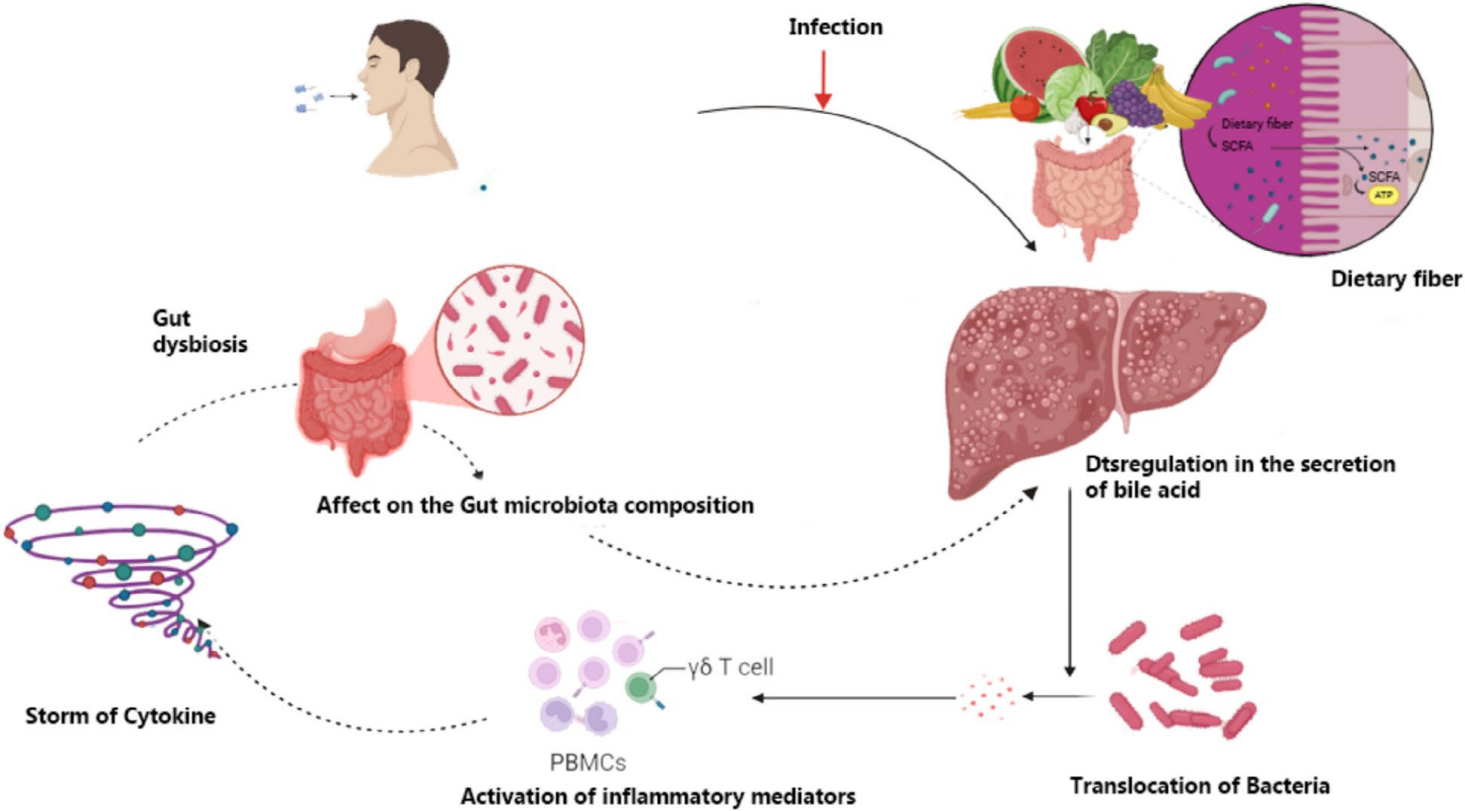
Pathophysiology of sepsis.

Both the hyperimmune and immunosuppressive phases of sepsis are marked by a sequence of immune responses that are triggered by the activity of different immunological cells. The host experiences sepsis as a result of an overabundance of inflammation and inhibition of some immunological responses, rather than launching an efficient antiviral immune response upon pathogen invasion (Delano and Ward 2016). The systemic activation of the innate immune system during the initial hyperinflammatory phase causes a severe and long‐lasting inflammatory response called the “cytokine storm,” which can be described by a significant production of inflammatory cytokines like IL‐1, TNF, and IL‐17. At this stage, neutrophils are more prevalent, and dendritic cells, lymphocytes, and macrophages are all stimulated. Damaged blood vessels may enable pathogens to travel throughout the body, triggering an extreme inflammatory response that results in systemic immunological dysregulation and damage. As a result, the generation of immune cells, the survival of effector cells, and their function are all immediately influenced, leading to widespread immunosuppression. Immunosuppression triggered by sepsis can be attributed to both innate and acquired immune dysfunctions, which are typified by excess production of immune regulatory cells, unchecked cellular apoptosis, the release of anti‐inflammatory cytokines, compromised immune cell function, T cell exhaustion, and restricted pro‐inflammatory gene transcription (Fu et al. 2023). Endotoxin tolerance, immune cell degradation, or cell anergy are all tightly linked to immunosuppression in sepsis. Impaired expression of human leukocyte antigen‐DR (HLA‐DR) and increased expression of immune checkpoint molecules, including B and T lymphocyte attenuator (BTLA), T cell immunoglobulin and mucin domain‐containing protein‐3 (TIM‐3), and programmed cell death protein 1 (PD‐1), worsen immunosuppression (Delano and Ward 2016). A number of variables, such as co‐morbid conditions, the amount of bacteria inoculum, and the pathogen's aggressiveness, affect the reaction of the immune system in sepsis (Figure 2).

## 5 Therapeutic Effects of Probiotics in Sepsis Management

Sepsis and critical illness are systemic illnesses that create a hostile gut environment that promotes pathogenic species by upsetting the intestinal microbiota. Defined as the “motor” of the systemic inflammatory response, the gut is a key player in the pathophysiology of sepsis. When intestinal epithelial homeostasis is disrupted during sepsis, proinflammatory cytokine production rises, the barrier malfunctions, and apoptosis increases, all of which can end in multiple organ failure. The fatality rate from sepsis is significant, and the present treatments for the condition are poor (Dos Santos et al. 2021). Finding novel therapeutic approaches that lower the disease's death rate is crucial. Finding new, effective drug agents is still necessary in spite of the widespread application of antibiotics and their synthetic counterparts to treat sepsis. This is because antibiotics have a number of unfavorable side effects, and in recent years, the rise in antibiotic‐resistant pathogens has raised serious concerns within the healthcare system. Probiotic therapy is one potential treatment plan for nosocomial infections in critical care units, which frequently result in sepsis and multiorgan failure (Davison and Wischmeyer 2019). Beneficial living bacteria called probiotics reside in the gastrointestinal system and regulate the immunological and inflammatory responses. Numerous investigations have demonstrated its therapeutic roles in treating severe infections in patients admitted to the critical care unit and preventing sepsis. Probiotics' biological properties have been investigated recently in an effort to extend their range of therapeutic and preventative modulation in both humans and animals (Khailova et al. 2013). These live microbes can give sick and immunocompromised people quantifiable physiological benefits if given in sufficient quantities.

### 5.1 Influence of Probiotics on Microbiota Homeostasis

There is strong evidence from current research, both in vitro and in vivo, that taking probiotics in adequate amounts offers a strong resistance against opportunistic and pathogenic microbes. Probiotics can help the gastrointestinal tract's weakened natural flora, which frequently occurs as a result of changes in eating habits, medical treatments, and the use of medication to treat intestinal illnesses (Kothari et al. 2019). Probiotics regulate microbial homeostasis through a number of mechanisms. First, probiotics improve the general structure of the human gut microbiota by boosting the number of intestinal good bacteria either by growing themselves or by encouraging the expansion of naturally occurring desirable microbial species. Second, to preserve intestinal equilibrium, probiotics use competitive exclusion (Dos Santos et al. 2021). A natural mechanism of competition for resources and ecological niches, competitive exclusion aids in the colonization of beneficial gut bacteria and inhibits the colonization of harmful bacteria. Thirdly, by regulating metabolite excretion and the intestinal environment, probiotics promote microbiota balance.

**FIGURE 3:**
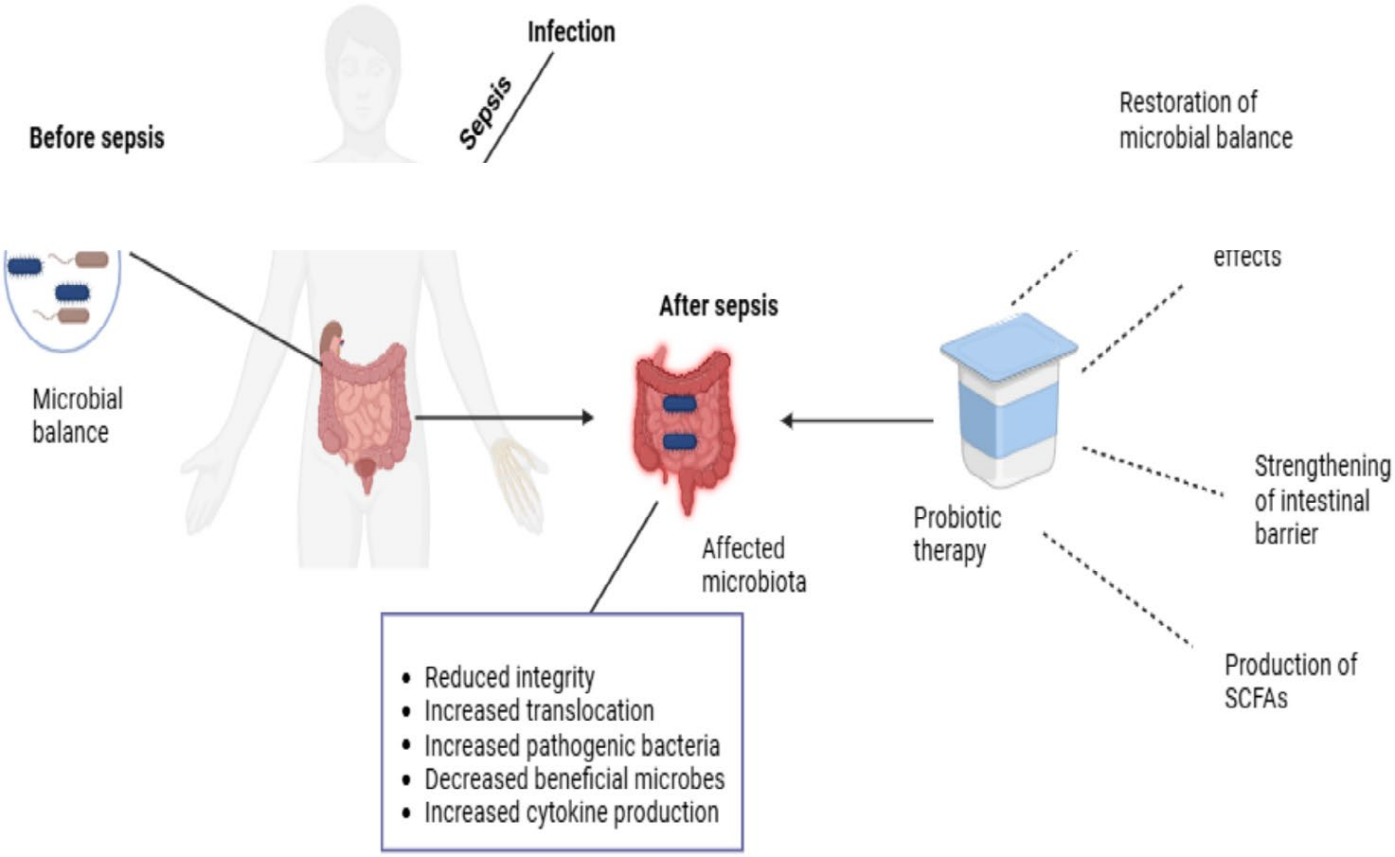
Potential role of probiotics in sepsis.

To explore how the intestinal barrier and microbial change occur in early sepsis and how a concomitant probiotic medication improved dysbiosis in the early stages of the illness, a randomized, double‐blind, placebo‐controlled pilot study was undertaken. The results revealed that a probiotic treatment does, in fact, result in more bacterial species and a higher level of functional diversity in feces. Sepsis with early onset alters the composition and function of the microbiome. Probiotic treatment was an effective technique to treat sepsis immediately because it could effectively manage microbiota, as demonstrated by Stadlbauer et al. (2019). In a different study, researchers sought to understand in detail how probiotic supplements affected the gut microbiota of critically sick septic patients during their initial ICU stay. According to Mahmoodpoor et al. (2022), the study showed that the probiotic preparation had a remarkable effect on the intestinal microbiota in critically sick septic patients by reducing the number of harmful bacteria. Another study showed that the helpful Prevotellaceae disappeared and the pathogenic *Staphylococcaceae* and Enterococcaceae appeared once sepsis began. Higher serum levels of cytokines that promote inflammation (IL‐22, IL‐2, TNF‐α, and IL‐6), epithelial cell apoptosis, and the breakdown of tight junctions were all indicators of worsening immune responses during sepsis, which have been connected to a high relative number of potentially pathogenic commensals, including Enterobacteriaceae, Bacteroidaceae, Erysipelotrichaceae, Deferribacteraceae, Clostridiaceae, and Pseudomonadaceae. Opportunistic microbes were reduced or eliminated with *Lactobacillus rhamnosus* GG (LGG) pretreatment, whereas helpful bacteria like Verrucomicrobiaceae proliferated, epithelial cell apoptosis diminished, and cell tight junction development and proliferation were fostered. According to Chen et al. (2019), the current findings imply that prophylactic LGG therapy may be beneficial for lowering sepsis mortality through restoring the balance of altered gut flora, regulation of systemic inflammation, and protection of mucosal barrier functionality (Figure 3).

Another study used LGG prophylaxis to investigate the potential therapeutic impact on host co‐microbiota and metabolism of sepsis‐induced dysbiosis in the colon microbiota of mice. The results demonstrated that LGG therapy addressed the gut microbiota imbalance caused by sepsis and greatly reduced sepsis death rates. In particular, LGG suppressed conditional detrimental bacteria including Proteobacteria and Deferribacteres, LPS‐producing Enterobacteriaceae, and facultative anaerobes such as Bacteroidaceae and Erysipelotrichaceae. Furthermore, it brought about an increase in organisms capable of producing energy, including Firmicutes (Chen et al. 2020). Mice have been utilized for researching the effects of sepsis and *S. thermophilus* on microbial community organization and the anti‐inflammatory properties of *S. thermophilus* . The findings suggest that *S. thermophilus* 19 may alter the gut microbiota while restoring equilibrium after a disruption brought on by sepsis.

### 5.2 Modulation of the Immune System

Probiotics have the potential to significantly impact the body's immune system by deliberately causing immune cells such as DCs, lymphocytes, macrophages, mast cells, granulocytes, or intestinal epithelial cells to release cytokines, such as chemokines, ILs, IFNs, TGFs, and TNFs. A study assessed how probiotics influenced the levels of cytokines in critically ill young children suffering from deadly sepsis when children who had severe sepsis were given probiotic supplements, their levels of proinflammatory and anti‐inflammatory cytokines substantially decreased (Angurana et al. 2018). Another study used a laboratory model of septic peritonitis to assess the influence of *Bifidobacterium longum* (BL) and LGG on intestinal epithelial balance, translocation of bacteria, death rates, and the response to inflammation. Probiotics brought colonic proliferation back to sham levels and dramatically decreased markers of colonic apoptosis. In contrast with septic animals, probiotics significantly decreased the expression of inflammatory cytokines in the colon and throughout the body. It was discovered that giving probiotics to children with experimental sepsis at the beginning of the illness can increase their chances of survival. Attenuation of the local and systemic inflammatory response and prevention of systemic bacteremia, potentially through higher intestinal epithelial equilibrium, are the mechanisms behind this defense (Khailova et al. 2013).

A study examined the effects of probiotic pretreatment on oxidative damage, antioxidant activity, inflammation, hematological parameters, and mortality in ill rats. The results indicated that septic rats given VSL #3 had lower concentrations of the cytokines that cause inflammation IL‐6, TNF‐α, IL‐10, and NF‐κB. It came to the conclusion that probiotic VSL #3, which improved antioxidant and anti‐inflammatory states and prolonged the survival of septic rats, could potentially be regarded as a novel complementary therapeutic method for treating sepsis (Chen et al. 2024).

Another study employed a mouse sepsis model and found that *S. thermophilus* 19 changes the host fecal microbiota and the inflammatory response produced by LPS. As demonstrated by the data, strain 19 was able to successfully repair the increased expression of TNF‐α, IL‐1β, and IL‐6 prompted by LPS treatment. This may well be because of a shift in the composition of the gut microbiota, most importantly the rate of phylum Fusobacteria species, according to Han et al. (2020). A recent study found that of the three probiotic‐derived BEVs, probiotic bacterial LGG‐derived BEVs had the most powerful impacts on triggering macrophage phagocytosis and intracellular bactericidal activity. As of right now, it is known that sepsis patients have reduced phagocytosis in their macrophages, which results in inefficient infection control (Zhu et al. 2024). The leftover pathogens may therefore result in a chronic local infection that could eventually lead to systemic inflammation. In actuality, even with the right antibiotic treatment, almost 80% of sepsis patients still have an unresolved septic focus when they pass away. Treatment approaches that increase bactericidal activity and macrophage phagocytosis may therefore be very promising for increasing patient survival. An investigation has been carried out to assess the possibility of therapeutic benefits of probiotic bacteria‐released extracellular vesicles (BEVs) in mitigating organ damage and death caused by sepsis. The findings indicated that BEVs produced by probiotics LGG activate the signaling pathway mediated by FPR1/2, which facilitates the enhancement of macrophage phagocytosis and bacterial clearance. According to Zhu et al. (2024), LGG‐derived BEVs probiotics have therapeutic potential for sepsis while providing better chances of survival. Besides, the probiotics are now recognized to regulate the host's immunological response through the effects exerted by the short‐chain fatty acids (acetate, propionate, and butyrate). Amazingly, SCFAs modulate inflammation by inhibiting immune cell‐mediated inflammatory cytokine production by cells including T cells, B cells, dendritic cells, neutrophils, and macrophages (Yao et al. 2022). SCFAs were recently reported to inhibit pro‐inflammatory mediators, including TNF‐α, IL‐6, and NO, which are synthesized by cytokines and LPSs. Several studies have reported that there is a decrease in the levels of SCFAs in patients with sepsis. Therefore, there may be a need to place more emphasis on SCFAs for the management of patients with sepsis in the ICU.

### 5.3 Strengthening of Intestinal Barrier Integrity

The intestinal barrier is an impermeable barrier that permits immune detection and the absorption of essential nutrients while keeping out germs and harmful chemicals. The host's systemic circulation may be highly susceptible to inflammatory agents, cytokines, and microbial subcomponents, especially LPS, due to impairments in barrier integrity (Guo et al. 2019). Sepsis occurs due to a disruption of the intestinal barrier. Different probiotic strains have been proved in several trials to maintain the structural integrity of the intestinal barrier. It has been determined that probiotics, such as *Escherichia coli* Nissle 1917 (EcN), help with a variety of gastrointestinal illnesses and preserve intestinal barrier integrity. A septic mouse model created via cecal ligation and puncture (CLP) surgery has been utilized in one investigation to examine the possible protective effect of EcN on intestinal barrier function. The results show that EcN promotes the strength of the intestinal barrier during sepsis by blocking NF‐κB‐mediated activation of the MLCK‐P‐MLC signaling pathway and by increasing both the amount and location of damaged tight junction (TJ) proteins (Guo et al. 2019).

Another study examined the preventive benefits of LGG, an essential probiotic that protects the wall of the gut during sepsis. Yılmaz and Erdem (2020) found that probiotics LGG are effective and affordable medicinal options that also enhance the immune system and decrease BT. Another study demonstrated the potential effect of live probiotic medication on improving the survival rate of experimental septic mice. According to Chen et al. (2016), improved function was associated with better gut barrier integrity, reduced ileum mucosal injury, and changed global blood metabolic profiles. Another research investigation found that oral administration of LGG to septic mice boosted their survival rate. It illustrated the protective properties of oral probiotic consumption during sepsis by indicating that LGG encourages intestinal stem cell (ISC) regeneration and supports an intestinal barrier weakened by sepsis.

## 6 Limitations and Recommendations

Notwithstanding the fact that administering a certain quantity of probiotics may benefit individuals suffering from a number of ailments, including sepsis, there are certain safety issues, especially when it comes to critically ill patients, the elderly, and infants. Probiotic translocation may result in allergic reactions, harmful immunological consequences, opportunistic systemic and local infections, and the spread of antibiotic resistance. In immunocompromised adults and young children, probiotic treatment may infrequently result in fungemia (Aydoğan et al. 2021). Probiotics could lead to bacteremia alongside fungemia, particularly in vulnerable people (Mikucka et al. 2022). Numerous studies have correlated the delivery or intake of probiotics to localized infections in various body areas, such as abscess and endocarditis (Ioannou et al. 2023). Probiotic supplements may induce cytokine creation, which may have significant immunological effects, including autoimmune diseases or particular inflammations. Thus, it is important to pay attention to how probiotics affect the progression of autoimmune diseases (Doron and Snydman 2015). Furthermore, probiotics may cause high‐risk individuals and children to become more sensitized (IgE) (Peldan et al. 2020). Although the majority of probiotic strains of Lactobacillus, Lactococcus, and Bifidobacteria are harmless and present no deadly threat, undesirable exceptions, such as the horizontal transfer of antibiotic resistance genes to pathogenic microbes that share the gut microbiome, may have major clinical implications (Imperial and Ibana 2016). Though these substances must be used with caution, probiotics have demonstrated advantages in the prevention and treatment of a number of disease situations. Numerous illness states have been proven to benefit from the use of probiotics in both prevention and treatment; nevertheless, these substances must be used with caution. Since not all critically ill patients are suitable candidates for probiotic therapy, hazards and advantages should be evaluated for each patient. Given the available data, probiotic usage for the treatment of sepsis should be used with caution, particularly in young children and the elderly. For upcoming in vivo investigations, we advise including the antibiotic group. Clinical trials that are large, powerful, and well‐designed are required, particularly those that target particular probiotic strains and subsets of sepsis patients, including pediatric patients.

## 7 Conclusion

The flora makeup and intestinal ecosystem of patients with sepsis undergo substantial alterations. Sepsis disrupts the intestinal mucosal barrier because it creates a disparity in the gut's local immune system and a surplus of gut flora. Intestinal flora translocation will be accelerated by intestinal barrier degradation, which will worsen sepsis. Management with probiotics may aid critically sick individuals in maintaining their disordered gut flora and lower the likelihood of infection consequences. In order to minimize complications related to infection and modulate immunity, probiotic supplementation may be a significant therapeutic alternative. The prospective role of probiotics as immunologic adjuvants that control the immune response and sepsis‐induced immunosuppression requires more investigation.

## Author Contributions

**Zhaopeng Wang:** validation (equal), visualization (equal), writing – original draft (equal). **Jiaqi Huang:** data curation (equal), writing – review and editing (equal). **Peng Zhao:** supervision.

## Ethics Statement

The authors have nothing to report.

## Consent

The authors have nothing to report.

## Conflicts of Interest

The authors declare no conflicts of interest.

## Data Availability

Even though adequate data has been given in the form of tables and figures, all authors declare that if more data is required, then the data will be provided on a request basis.
